# Trust in health information sources and its associations with COVID-19 disruptions to social relationships and health services among people living with HIV

**DOI:** 10.1186/s12889-021-10856-z

**Published:** 2021-04-28

**Authors:** Seth C. Kalichman, Bruno Shkembi, Moira O. Kalichman, Lisa A. Eaton

**Affiliations:** Institute for Collaborative Health Intervention and Policy, University of Connecticut, 2006 Hillside Road, Storrs, CT 06269 USA

**Keywords:** COVID-19, HIV infection, Trust, Health information

## Abstract

**Background:**

SARS-CoV-2 infection (COVID-19) is potentially severe for individuals with compromised immune systems, including people living with HIV. Along with the direct health threats of COVID-19, there are disruptions to social relationships and health services resulting from mitigation efforts instituted by public health authorities. This study examined the relationship between trust in the government and trust in COVID-19 health information from the US CDC, state health departments, and social media on the experience of COVID-19 social and health services-related disruptions.

**Methods:**

People living with HIV (*N* = 459) recruited through social media advertisements and chain referrals completed confidential surveys delivered through an online platform.

**Results:**

Participants experienced high-levels of disruptions to social relationships and health services attributable to COVID-19 mitigation efforts. We also observed high-rates of inaccurate information and low-levels of trust in government and sources of COVID-19 information. Greater disruptions to social relationships were predicted by more concern about oneself and others contracting COVID-19, whereas disruptions to health services were predicted by greater concern for oneself contracting COVID-19, greater general medical mistrust, and less trust in information from the CDC.

****Conclusion**s:**

Findings have implications for the necessity of rebuilding public trust in credible sources of health information and stepping up efforts to counter sources of inaccurate information.

**Supplementary Information:**

The online version contains supplementary material available at 10.1186/s12889-021-10856-z.

## Background

The SARS-CoV-2 pandemic is a public health crisis unparalleled in modern times, posing significant health threats to individuals with underlying chronic conditions [1–3]. While the nature of the various underlying conditions that determine severity of SARS-CoV-2 disease (COVID-19) are not fully understood, it is established that conditions that compromise the immune system raise concerns for COVID-19 severity, including HIV infection [4, 5]. In the UK, for example, people with HIV are more than twice at risk of COVID-19 death compared to people without HIV [6]. The vulnerabilities to COVID-19 that occur with HIV infection are countered by the potential protective effects of antiretroviral therapy (ART) as well as HIV-related changes to the immune system [7, 8].

In addition to the obvious direct health threats of COVID-19, there are indirect impacts of the COVID-19 pandemic, particularly ramifications of stay-home orders and physical distancing taken to mitigate the spread of the virus. In March 2020, more than a month following the first death from COVID-19 in the US [9], the Centers for Disease Control and Prevention (CDC) issued recommendations to avoid social gatherings but did not institute a national strategy for stemming infections [10]. Without a national plan for managing the COVID-19 outbreak [11], state and local health departments were required to formulate strategies to mitigate the spread of the virus. The City of Atlanta, for example acted to issue protective orders including invoking a state of emergency and closing all non-essential businesses [12], which did not however include health service providers. Reports from HIV clinical settings indicated the potential for interruptions to essential HIV care services despite remaining open [13]. Subsequent research has shown that measures taken to mitigate the spread of SARS-CoV-2 did disrupt health services for some people living with HIV by impeding access to medications and having to reschedule health service appointments [14, 15]. In addition to disruptions in health services, efforts to contain COVID-19 have adverse impacts on social relationships [16, 17], potentially increasing isolation, stress, and depression [16].

COVID-19-related disruptions to social relationships and health services occur in a context of public health messaging, including messages from health authorities as well as informal social networks. The CDC and state health departments, all government entities, have traditionally played crucial roles in delivering public health information, especially during health crises such as influenza outbreaks and acts of bioterrorism [18, 19]. In the era of COVID-19, however, trust in the CDC and health departments may have been eroded by conflicting information from government officials, including the United States President, the CDC leadership, and state health departments [20]. The influence of public health messages depends in part on the degree of trust ascribed to the messenger. Trust is a complex emotional state that is considered essential to public health messaging [21, 22]. In health communications, including research on responses to the 2009 H1N1 pandemic, trust is characterized by perceived credibility, which is a function of multiple elements including expertise, concern and care [19]. Unfortunately, trust in health communications from government sources has eroded in recent years [18, 23].

Lack of government trust is known to undermine public health messaging [23] and COVID-19 health messaging may be especially harmed by its politicized messaging [24]. The spread of misinformation and disinformation through multiple channels has undermined public health trust which has negatively impacted COVID-19 containment and mitigation efforts [25]. Deleterious effects of inaccurate information have undermined previous public health crises as seen in HIV denialism, where embracing HIV conspiracy theories and disregarding evidence-based HIV prevention interventions has had disastrous consequences in South Africa, Russia and the United States [25–27].

In addition to governmental platforms, social media outlets have become a significant source for COVID-19 information [28]. Inaccurate COVID-19 information has rapidly spread through social media platforms [29], with the potential to influence behavior [30]. Inaccurate information can fuel both a disregard for public health recommendations and a further distrust in health services [31]. In a study conducted in the US in April, 2020, Fridman et al. [32] found that trust in government sources of information, such as the CDC, was positively associated with accurate knowledge about COVID-19 and adherence to recommended mitigation strategies. Conversely, trust in private sources of information, including social media, was negatively associated with accurate knowledge about COVID-19 and adherence to recommended mitigation strategies. The degree to which people living with HIV trust COVID-19 information sources and the impact trust may have on the experience of disruptions to social relationships and health services has not yet been reported.

The current study was guided by previous research on trust in information sources and protective behaviors [18, 19]. We examined relationships among  COVID-19-related trust in the government’s response to COVID-19, trust in COVID-19 information from the CDC, state health departments, and social media with disruptions to social relationships and health services. Because social media outlets are unfiltered and informal, they have become common sources for inaccurate COVID-19 information [28], and may be related to consequences that occur when guidelines are not followed -- fewer disruptions in social relationships and greater avoidance of health services. On the other hand, greater trust in official sources of COVID-19 information, such as the CDC and state health departments, may be related to consequences that occur from following guidelines -- greater disruptions to social relationships and continued engagement in health services.

To examine these associations, we conducted a rapid-response survey with men and women living with HIV, a population vulnerable to more severe COVID-19 disease processes. The study aimed to test a model positing trust in information sources as predictors of disruptions to social relationships and health services among people living with HIV over and above the potential influences of demographic characteristics, information exposure, COVID-19 knowledge, and perceived risk (i.e., concern) for contracting COVID-19 and general medical mistrust. We conceptualized COVID-19 disruptions as the indirect impacts of the pandemic on people’s lives, which may stem from personal decisions to quarantine and socially distance as well as actions taken by others to reduce the spread of COVID-19. Our approach was to use hierarchically ordered regression models to control for potential confounds while also examining their independent effects. We hypothesized that greater trust in social media would be associated with fewer disruptions to social relationships and greater disruptions to health services over and above the other variables included in the model. In contrast, we hypothesized that greater trust in the government, the CDC and the state health department would be associated with greater disruptions to social relationships and fewer disruptions to health services.

## Methods

### Participants

Participants in the current study were men and women living with HIV in the state of Georgia, with more than 80% residing in the Atlanta metropolitan area. Participants were recruited through social media outlets and snowball chain referrals. Eligibility criteria included age 18 and older, African American / Black, and HIV positive status confirmed using video chat by showing a photo identification and a name-matching HIV test result, HIV viral load report, or ART medication bottle.

### Procedures

Following a phone conducted enrollment interview and informed consent, participants were sent a link to complete a self-administered survey. Surveys were delivered in two parts to reduce time burden. Part 1 included measures of demographic and health characteristics, substance use, HIV stigma, perceptions and medication beliefs. Part 2 included the measures of COVID-19 experiences, COVID-19 behaviors, knowledge and perceptions, trust in COVID-19 information, mental health symptoms, medical mistrust and perceptions of community connectedness. The average time participants took between the two parts was 44 min. All of the central measures in this study were included in Part 2. Data collection occurred between April 13 and August 30, 2020. The University Institutional Review Board approved all study procedures. All measures were administered through the Redcap electronic survey delivery system and included as supplementary material (file name Kalichman_HIV_COVID_SURVEY.pdf).

### Measures

#### Demographic and health characteristics

Participants reported their basic demographic information, including gender, age, marital status, race, education and income. We also asked participants whether they were currently receiving HIV care, including ART, and their most recent HIV viral load. Participants were asked whether they had heard about COVID-19, whether they believe they have had COVID-19, whether they were tested for COVID-19 and the results of their test.

#### COVID-19 knowledge

We asked seven questions regarding COVID-19 that had correct and incorrect answers. Items were derived from information available at the World Health Organization’s public information webpage [33]. Exact items are shown in the results section and were responded to as True, False or I Don’t know. Correct responses were scored 1 and incorrect and do not know responses were scored 0. Correct responses were summed to create a composite score (Cronbach’s α = .87). Participants also reported how many hours they estimate of daily online / smartphone use as an indicator of potential exposure to online information.

#### COVID-19 perceived vulnerability

We assessed participant concern that they may contract COVID-19 using a 100-point rating scale in response to the question: “From 0 to 100, how concerned are you about catching COVID-19”, with 0 = not at all concerned and 100 = extremely concerned.” The item was repeated for concern about others, specifically “From 0 to 100, how concerned are you about someone you know catching COVID-19?” using the same response format. Responses used a slide-bar tool where participants tapped on an anchored continuum.

#### Medical mistrust

Participants completed an adapted version of the Medical Mistrust Index [34]. The items reflect a sense of dishonesty and deception in the medical system. This scale contained 8-items selected on the basis of non-redundant item content, including “Health care providers have sometimes done harmful things to patients without their knowledge” and “Patients have sometimes been deceived or misled by health services providers”. Responses were on a 6-point scale, 0 = Strongly disagree to 5 = Strongly agree (Cronbach’s α = .87).

#### Trust in Government’s COVID-19 response

Participants were asked how much they trust that the government is doing all it can regarding COVID-19. Responses were made on a 4-point scale, 0 = Not at all trust to 3 = Completely trust (see Results for exact item).

#### Trust in Sources of COVID-19 information

Participants were asked how much they trust three sources for COVID-19 information; trust in information from the CDC, the state department of public health and social media. Responses were made on a 4-point scale, 0 = Not at all trust to 3 = Completely trust (see Results for exact items).

#### COVID-19 social relationships disruptions

Participants reported whether they had experienced four disruptions to social relationships as a result of COVID-19. Social relationship disruptions focused on canceling plans to be with others, being told not to work or go to school, and avoiding public transportation. The disruptions were responded to using three options to indicate whether each disruption had been experienced: 0 = No, 1 = Yes, a little, and 2 = Yes, a lot. We formed an index of COVID-19 social relationship disruptions by summing responses to the four social disruption items.

#### COVID-19 health services disruptions

Participants also reported whether they had experienced five disruptions to health services as a result of COVID-19, including being unable to go to the pharmacy, being unable to access medications and health services providers cancelling appointments. These disruptions were also responded to using three options to indicate whether each disruption had been experienced: 0 = No, 1 = Yes, a little, and 2 = Yes, a lot. The five health services disruptions were also summed to create an index score.

### Data analyses

We first report the sample characteristics, including COVID-19 knowledge, social relationships and health services disruptions, and responses to the trust measures. To describe the sample, we grouped participants using a median of COVID-19 disruptions, including social relationships and health services disruptions. For the descriptive analyses we grouped participants with lower disruptions (≤7, *N* = 242) and higher disruptions (> 8, *N* = 217). Descriptive analyses were performed using contingency table *X*^2^ tests for categorical variables and independent *t*-tests for continuous measures. To examine the relative differences in trust ascribed to government, the CDC, state health department, and social media we conducted within-subjects dependent t-tests. We also report the bivariate associations using Pearson correlation coefficients among variables included in the main analyses.

For the main analyses, we performed two separate hierarchical regression analyses predicting: (a) COVID-19 social relationship disruptions and (b) COVID-19 health services disruptions. Predictor variables were entered in a sequence of five conceptually ordered blocks: (a) Demographic characteristics: age, gender (coded 0 = male, 1 = female), and years of education, (b) COVID-19 information exposure: COVID-19-related knowledge and smartphone screen time, (c) Perceived vulnerability: COVID-19 concern for self and others, (d) Medical mistrust, and (e) Trust in government response to COVID-19 and trust in three information sources: the CDC, the state health department, and online / social media outlets. The blocks of variables allow for interpreting the unadjusted associations of variables to the outcome measure. Conceptually, the order was based on entering factors distal to trust first (demographics, information accuracy and exposure) followed by motivational factors (concern, medical mistrust), and finally entering trust in government and COVID-19 information sources. The five blocks of predictor variables were entered hierarchically such that all variables in previous models were carried forward and controlled in subsequent models. All statistical tests defined significance as *p* < .05.

## Results

Participants were 369 men and 90 women living with HIV. Ninety-seven percent of participants were African American, 62% had attended at least some college, and the average age for the sample was 34.3 years (SD = 9.3, range 20 to 66). There were no differences between participants experiencing less or greater COVID-19 related disruptions on demographic and health characteristics (see Table 1).

**Table 1:** Participant characteristics and COVID-19 experiences among people living with HIV experiencing less or greater COVID-19 related disruptions

	Less COVID-19 Disruptions *N* = 242		Greater COVID-19 Disruptions *N* = 217			
	N	%	N	%	*X*^2^	t
Men	199	82	170	78	1.1	
Women	43	18	47	22		
African American	236	97	210	97	0.5	
Age in years [M, SD]	34.3	8.8	34.7	9.6		0.4
Hours smartphone screen time [M, SD]	5.6	1.5	5.5	1.6		0.6
Education						
Less than high school	13	5	19	9	2.9	
Graduated high school	70	29	65	30		
At least some college	159	66	81	61		
Currently unemployed	80	33	73	34	1.3	
Married	16	7	15	7	4.3	
Currently in HIV care	230	95	207	95	0.1	
Currently taking antiretroviral therapy	228	94	208	96	0.6	
Most recent HIV viral load						
Detectable	14	6	15	7	0.5	
Undetectable	206	90	181	90		
Does not know	10	4	11	2		
Believe they may have had COVID-19	49	20	34	16	1.6	
Has been tested for COVID-19	71	29	88	41	6.3**	
Received positive COVID-19 test result	6	8	9	10	0.6	
Concerned about self COVID-19^a^ [M, SD]	61.5	33.8	80.4	26.2		6.6**
Concerned for others COVID-19^a^ [M, SD]	70.6	30.4	83.6	22.7		5.1**
**COVID-19 Social Disruptions**^**b**^						
You asked others to stay away to avoid getting COVID-19.	148	61	208	96		
You have been asked by others to stay away to protect you from getting COVID-19.	149	62	215	99		
You were told not to come to work or school because of COVID-19.	102	42	177	82		
Avoided public transportation because of COVID-19.	125	52	206	95		
**COVID-19 Healthcare Disruptions**^**b**^						
Been unable to get to a pharmacy because of COVID-19.	22	9	114	53		
Been unable to get to medicine you need because of COVID-19.	16	7	103	48		
You cancelled a clinic or doctor because of COVID-19.	41	17	128	59		
A clinic or doctor closed or cancelled your appointment because of COVID-19.	95	39	184	85		
A service provider of any type closed or cancelled your appointment because of COVID-19.	98	41	181	83		

Note: ^a^ 100-point rating scale, 0 = not at all concerned to 100 = extremely concerned; ^b^ Disruptions dichotomized responses for any occurrence by collapsing ‘a little’ and ‘a lot’ responses; *X*^2^ tests for social and healthcare disruptions are not shown due to circularity; * *p* < .05, ** *p* < .01

### COVID-19 social relationship disruptions

The frequencies of COVID-19 social relationship disruptions are shown in Table 1. Each of the disruptions to social relationships were reported by at least half of participants. More than 60% of participants asked others to stay away and were asked by others to stay away to protect against COVID-19. In addition, more than 70% of the sample indicated that they avoided using public transportation to protect against COVID-19.

### COVID-19 health services disruptions

The frequencies of COVID-19 health services disruptions are also shown in Table 1. More than 60% of participants had experienced medical as well as other service provider appointment cancellations due to COVID-19. In addition, more than one in five participants indicated being unable to get to a pharmacy and unable to access medications because of COVID-19.

### COVID-19 awareness, concern, and knowledge

All participants had heard of COVID-19 at the time of the survey, with 18% (*n* = 83) believing they may have had COVID-19 and 35% (*n* = 159) having been tested, with those who had greater disruptions being more likely to have been tested. A total of 9% (*n* = 15) of participants who had been tested received a positive result. In addition, participants with greater COVID-19 disruptions indicated greater concern for themselves and others contracting the virus (see Table 1). With respect to COVID-19 knowledge, participants on average answered 4 of the 7 questions correctly; one in four participants did not know that the virus that causes COVID-19 is new to humans, half of participants believing that the viral infection can be cured with antibiotics, and over 40% believed that eating garlic offers protection against the virus. COVID-19 knowledge did not differ between participants with lower and higher COVID-19 disruptions (see Table 2).

**Table 2:** COVID-19 related knowledge and trust in information sources among people living with HIV experiencing less or greater COVID-19 related disruptions

	Less COVID-19 Disruptions *N* = 242		Greater COVID-19 Disruptions *N* = 217			
	N	%	N	%	*X*^2^	t
**COVID-19 Related Knowledge**^**a**^						
Antibiotics can cure coronavirus. [F]	132	55	101	46	2.9	
People of all ages can become infected with the coronavirus. [T]	232	96	203	94	1.2	
Coronavirus has been around a long time and only recently came to the USA. [F]	64	26	52	24	0.3	
Eating garlic can lower your chances of getting infected with the coronavirus. [F]	106	43	95	43	0.1	
Coronavirus is no different than a bad case of the flu. [F]	126	52	117	54	0.2	
Most people who are infected with the coronavirus recover from it. [T]	156	65	117	54	5.2*	
Antibiotics can be used to prevent infection from the coronavirus. [F]	109	45	81	37	2.8	
The coronavirus can be cured with a drug used to treat Malaria.[F]	95	39	90	41	0.2	
COVID-19 Related Knowledge [mean, SD]	4.2	2.2	3.9	2.2		1.2
**Trust in Government**^**b**^						
How much do you trust that the Government is doing all it can to prevent the spread of COVID-19?						
Completely trust	58	24	44	20	1.7	
Somewhat/slightly trust	67	27	71	33		
Not at all trust	117	48	102	47		
Mean [SD]	0.81	0.92	.78	0.88		0.3
**Trust in COVID-19 Information Sources**^**b**^						
How much do you trust information from the CDC about COVID-19?						
Completely trust	131	54	87	40	9.3**	
Somewhat/slightly trust	68	28	84	39		
Not at all trust	43	18	46	21		
Mean [SD]	1.55	0.99	1.35	0.92		2.1*
How much do you trust information from the State Department of Public Health about COVID-19?						
Completely trust	118	26	85	18	4.3	
Somewhat/slightly trust	81	34	82	38		
Not at all trust	43	18	49	23		
Mean [SD]	1.43	0.92	1.29	0.95		1.6
How much do you trust information you are seeing online or in social media about COVID-19?						
Completely trust	59	13	64	30	1.6	
Somewhat/slightly trust	106	44	90	42		
Not at all trust	77	32	62	13		
Mean [SD]	0.96	0.81	1.07	0.88		1.4

Note: ^a^ *F* false, *T* true, number and percent correctly responding; ^b^responses coded 0 = Not at all trust, 3 = Completely trust

### Trust in Government and COVID-19 information sources

Analyses of trust in government and sources of COVID-19 information are shown in Table 2. Results indicated that neither the government nor the various sources of health information were trusted completely by more than half of participants. Dependent t-tests performed among trust in government and the sources of health information are shown in Table 3. Results indicated that the government was trusted significantly less than all three sources of COVID-19 information. In addition, both the CDC and the state health department were trusted for COVID-19 information more than social media. Finally trust in the CDC was greater than the state health department.

**Table 3:** Mean trust ratings and within-subjects pairwise comparisons

Trust variables	Mean	SD	Dependent t-Tests
How much do you trust that the Government is doing all it can to prevent the spread of COVID-19?	0.79	0.89	CDC, t = 14.63, *p* < .01 State Health Dept., t = 13.22, *p* < .01 Online/Social Media, t = 4.98, *p* < .01
How much do you trust information from the CDC about COVID-19?	1.45	0.98	Online/Social Media, t = 9.64, *p* < .01 State Health Dept., t = 2.99, *p* < .01
How much do you trust information from the State Department of Public Health about COVID-19?	1.36	0.93	Social Media, t = 8.09, *p* < .01
How much do you trust information you are seeing online or in social media about COVID-19?	1.01	0.84	

### Bivariate associations

Table 4 shows the bivariate correlations among COVID-19 related disruptions, knowledge and concerns, medical mistrust, trust in government, and trust in the sources of COVID-19 information. Social relationship disruptions were significantly correlated with concern about contracting COVID-19 and someone else contracting COVID-19. COVID-19 disruptions to health services were significantly negatively correlated with COVID-19 knowledge and trust in the CDC for COVID-19 information, and positively correlated with concern about oneself contracting COVID-19 and medical mistrust. In addition, greater trust in government and trust in all sources of COVID-19 information were significantly negatively correlated with medical mistrust, where greater medical mistrust was related to less trust in the government, CDC, the state health department and social media.

**Table 4:** Pearson correlation coefficients for COVID-19 disruptions, COVID-19 knowledge, COVID-19 concern and trust in government and trust in sources of COVID-19 information

							Trust		
	Social Disruptions	Health Service Disruptions	COVID-19 Knowledge	Self-Concern	Others- Concern	Medical Mistrust	Government	CDC	State Health Department
Healthcare Disruptions	.49**								
COVID-19 Knowledge	−.01	−.13**							
Self-Concern	.32**	.20**	.02						
Others -Concern	.32**	.18**	.06	.76**					
Medical Mistrust	.02	.21**	−.18**	−.02	−.02				
Trust Sources									
Government	.03	−.01	−.05	−.01	.02	−.19**			
CDC	−.04	−.17**	.11*	.09	.13**	−.27**	.48**		
State Health Department	−.03	−.11*	.08	.09	.09	−.23**	.51**	.75**	
Social Media	.05	−.03	−.05	.08	.04	−.14**	.45**	.43**	.46**

Note: * *p* < .05, ** *p* < .01

### Predictors of COVID-19 disruptions to social relationships

Results of the multivariable regression models predicting COVID-19 disruptions to social relationships showed that age, gender, years of education, smartphone screen time, and COVID-19 related knowledge did not significantly predict social relationships disruptions, F(5,447) = 0.20, *p* > .1 (see Model 2, Table 5). Adding concern about oneself and others contracting COVID-19 in Model 3 did significantly predict social disruptions, F(7,445) = 9.26, *p* < .001, accounting for 12.7% of the variance. Medical mistrust did not significantly add to the predictive model or change the variance accounted for, although Model 4 remained significant, F(8,444) = 8.12, *p* < .001, *R*^2^ = .128. Finally, in Model 5, concern for self and others contracting COVID-19 remained significant predictors F(12,440) = 6.16, *p* < .001, accounting for 14.4% of the variance, and none of the trust variables added to the model.

**Table 5:** Standardized coefficients from hierarchical regression models of COVID-19 social relationship disruptions

Variables	Model 1	Model 2	Model 3	Model 4	Model 5
**Demographic characteristics**					
Age	−.004	−.007	−.060	−.060	−.067
Gender	.033	.030	.077	.078	.073
Years education	−.022	−.017	.035	.036	.055
**Exposure to information**					
COVID-19 Knowledge		−.011	−.029	−.026	−.011
Phone Screen-time		−.021	.004	.003	−.003
**Perceived vulnerability**					
Self-Concern about COVID-19			.210**	.211**	.206**
Concern for others about COVID-19			.178**	.178**	.200**
**General Medical Mistrust**					
Medical Mistrust				.021	.009
**Trust sources**					
Government					.087
CDC					−.121
State Health Department					−.037
Online / Social Media					.067
	F(3,449) = 0.25, *p* = .859 *R*^2^ = .002	F(5,447) = 0.20, *p* = .963 *R*^2^ = .002 *R*^2^ Δ = 0	F(7,445) = 9.26, *p* < .001 *R*^2^ = .127 *R*^2^ Δ = .125**	F(8,444) = 8.12, *p* < .001 *R*^2^ = .128 *R*^2^ Δ = .001	F(12,440) = 6.16, *p* < .001 *R*^2^ = .144 *R*^2^ Δ = .016

Note: *R*^2^ Δ = *R*^2^ change, ** *p* < .01

### Predictors of COVID-19 disruptions to health services

Results of the multivariable regression models predicting disruptions to health services are shown in Table 6. In the first model, age, gender, and years of education did not predict health services disruptions, F(3,449] = 2.32, *p* > .1. Adding COVID-19 knowledge and online / smartphone use improved the model, F(5,447) = 2.40, *p* < .05, with less COVID-19 knowledge significantly contributing to the model. In Model 3, COVID-19 knowledge remained significant and concern about oneself contracting COVID-19 significantly predicted health services disruptions, F(7,445) = 4.09, *p* < .01, *R*^2^ = .060. Medical mistrust also significantly added to the predictive value in Model 4, F(8,444) = 6.08, *p* < .01, *R*^2^ = .099, a significant increase of 3.9% of the explained variance, F = 118.8, *p* < .01. However, COVID-19 knowledge was no longer significant in Model 4. In the final model that included trust in information sources, greater self-concern in contracting COVID-19 and greater medical mistrust predicted greater disruptions in health services, and less trust in information from the CDC predicted greater health services disruptions, F(12,440) = 4.99, *p* < .01, *R*^2^ = .120, a significant addition of 2.1% to the explained variance, F = 2.62, *p* < .05.

**Table 6:** Standardized coefficients from hierarchical regression models of COVID-19 health services disruptions

Variables	Model 1	Model 2	Model 3	Model 4	Model 5
**Demographic characteristics**					
Age	.037	.053	.024	.032	.027
Gender	.059	.045	.072	.080	.070
Years education	−.091	−.066	−.035	−.026	−.003
**Exposure to information**					
COVID-19 Knowledge		−.110*	−.114*	−.081	−0.69
Phone Screen-time		.007	.029	.027	.018
**Perceived vulnerability**					
Self-Concern about COVID-19			.240**	.246**	.244**
Concern for others about COVID-19			−.070	−.073	−.048
**General Medical Mistrust**					
Medical Mistrust				.199**	.178**
**Trust sources**					
Government					.102
CDC					−.180**
State Health Department					.011
Online / Social Media					.011
	F(3,449) = 2.32, *p* = .075 *R*^2^ = .015	F(5,447) = 2.40, *p* = .036 *R*^2^ = .026 *R*^2^ Δ = .011	F(7,445) = 4.09, *p* < .001 *R*^2^ = .060 *R*^2^ Δ = .034**	F(8,444) = 6.08, *p* < .001 *R*^2^ = .099 *R*^2^ Δ = .039**	F(12,440) =4.99, *p* < .001 *R*^2^ = .120 *R*^2^ Δ = .021*

Note: *R*^2^ Δ = *R*^2^ change, * *p* < .05, ** *p* < .01

## Discussion

The current study was conducted 6-months into the US SARS-CoV-2 epidemic and all of the people living with HIV who participated were aware of COVID-19; one in three (34%) had been tested for COVID-19. The study results only partially confirmed our hypotheses. First, we did not confirm our hypothesis that greater trust in online / social media sources of information would be related to fewer social relationship disruptions and greater disruptions to health services. We did not identify significant associations between trust in online / social media information and COVID-19 outcomes in either bivariate or multivariable analyses. Although social media outlets have been found to propagate COVID-19 myths and misinformation [28, 29, 31] and previous research has shown that greater trust in private and social media outlets for COVID-19 information was associated with less adherence to social distancing guidelines [32], we did not find that trust in online / social media information sources was related to disruptions in social relationships or health services. One factor that may have influenced our findings is the race and age of our participants. Fridman et al. [10] found that white and younger persons are more likely to trust social media for COVID-19 information, whereas our participants were living with HIV, mostly people of color and over 30 years of age. Past studies on responses to the H1N1 pandemic also found important racial differences in trust of government sources of information, with minorities reporting greater trust in government information than whites [19]. These varied findings concerning demographic differences in trusting information sources regarding different public health crises at different times in history are important to consider when comparing study results.

We also did not confirm our hypothesis that trust in the CDC and the state health department for COVID-19 information would be associated with greater disruptions to social relationships. However, in bivariate associations we did confirm that greater trust in the CDC and the state health department were associated with fewer disruptions to health services. However, in the hierarchical regression models, only greater trust in in the CDC was significantly associated with fewer health service disruptions, adding 2.1% to the explained variance. Although the CDC has offered guidance for sustaining non-COVID-19 health services during the pandemic [35], interruptions in health services for people with HIV have been reported [2].

Participants demonstrated considerable inaccurate information about COVID-19. Despite the sample living with a critical underlying condition, more than 40% of participants did not know that COVID-19 cannot be prevented or cured by using antibiotics and cannot be cured using drugs that treat malaria. In addition, half of participants indicated that COVID-19 is no different than a bad case of the flu and 40% of participants believed that eating garlic can lower the chance of contracting COVID-19. This level of inaccurate information runs in parallel to mixed and often conflicting public health messaging from the US government and public health authorities. Most notably, President Trump questioning public health science, widely promoting conspiracy theories and falsely claiming an anti-malaria drug can treat COVID-19 has been a concern for eroding public trust in health messaging [36, 37]. Similar levels of COVID-19 inaccurate information have been reported in other studies [38, 39], including studies that find people who rely more on social media and social networks for information are less well-informed [40]. Although our study is unable to draw any causal connection between inaccurate information and lack of trust in the US government or COVID-19 information sources, we also cannot rule out such a connection.

The current findings should be interpreted in light of their methodological limitations. All of the data in this study was cross-sectional and none of the findings can be interpreted to mean directional or causal relationships. COVID-19 disruptions resulting from personal decisions as well as the acts of others may directionally influence information trust and vice versa. The sample for this study was one of convenience and cannot be considered representative of people living with HIV. In addition, the sample was mostly African-American men and therefore limited in its generalizability to women and people of various other ethnic/racial backgrounds. In addition, we are unable to know whether the results of this study would vary with a non-HIV positive sample. Future research is needed to replicate these findings with different populations of people living with HIV as well as the general population. Our approach to the regression analyses was also limited by not examining potential theoretically important patterns of association. For example, future research may examine whether accurate COVID-19 information mediates the relationship between trust in information sources and COVID-19 disruptions. It should also be noted that we recruited several participants from social media platforms which may have influenced the trust responses, particularly trust in social media. Although we controlled the amount of time using smartphones in the analysis, we did not control for use of other devices that can access a wide array of information. Our measures of social relationships and health services disruptions were not exhaustive and we did not measure individual behaviors that can be undertaken to reduce the spread of SARS-CoV-2, such as mask-wearing and physical distancing. The study was undertaken in the early months of the COVID-19 crisis and our findings may therefore be transient and specific to this time period. While the results have implications for future spikes in COVID-19 outbreaks and future pandemics, they may also be unique to this particular time period in this pandemic. Our study relied entirely on self-report instruments that are subject to social response biases. Nevertheless, these findings have implications for public health communications and messaging in response to COVID-19.

Our findings show that despite inaccurate information and distrust of sources of public health information, there was a high-degree of social distancing reflected by the social disruptions participants experienced. Efforts to provide correct information about COVID-19 should be intensified among people living with HIV to assure that the disruptions they endure are in fact consistent with mitigating viral transmission. That is, we cannot be sure that the social relationships and health services disruptions experienced did indeed mitigate COVID-19 risks. For example, although some social interactions may have been disrupted, we do not know what social interactions did occur and to what degree measures such as mask-wearing were used to mitigate risk. Efforts should be taken to restore trust in a confluence of health messages, including from the CDC, health departments and other government entities. Online platforms including social media afford opportunities to correct inaccurate information and promote preventive actions [41]. These platforms, which have often been the focus of spreading false information, including through the office of the US President, can be exploited to directly counter myths, conspiracy theories, and inaccurate information by credible health authorities. Previous research shows that trust in information coming from government authorities predicts acceptance of new vaccines [42]. Restoring trust through credible health information sources is increasingly urgent as SARS-CoV-2 preventive vaccines are available, making trusted communications vital in the fight against COVID-19.

## Conclusions

Accurate and trusted health information is essential to guiding the public toward disease prevention, screening and treatment. Public health authorities at the federal, state and local levels are uniquely situated to deliver accurate health information. However, lack of trust in the CDC and state health departments distracts attention and undermines adherence to health guidelines. People who are vulnerable to severe COVID-19 health outcomes, in this case people living with HIV, experience greater disruptions to their healthcare when they do not trust health authorities. Establishing trust in public health messaging targeted to priority populations is necessary to improve adherence to public health guidance without disrupting health services.

## Supplementary Information

**Additional file 1.** Survey instrument items in the current study

## Data Availability

All data and materials are available from the authors by request.

## Abbreviations

HIV: Human Immunodeficiency Virus
ART: Antiretroviral therapy
CDC: Centers for Disease Control and Prevention
