# Supplementary material for: Trust in health information sources and its associations with COVID-19 disruptions to social relationships and health services among people living with HIV
Source: BMC Public Health. 2021 Apr 28;21:817. doi: 10.1186/s12889-021-10856-z (PMC8080999; doi:10.1186/s12889-021-10856-z)
Supplement: Supplementary file 1 — Additional file 1. Survey instrument items in the current study [file 12889_2021_10856_MOESM1_ESM.pdf]

UConn Health
Research IT - PRODUCTION

KISS Study Health Survey Pilot

Codebook

Data Dictionary Codebook

10/29/2020 5:55pm

#	Variable / Field Name	Field Label<br>*Field Note*	Field Attributes (Field Type, Validation, Choices, Calculations, etc.)
Instrument: **Participants** (participants)			
1	participant_id	Participant ID	text, Identifier
2	phone	Phone	text (phone), Identifier
3	email	Email	text (email)
4	pcpt_gender	Participant is...	radio, Required<br>1 Male<br>2 Female
5	pcpt_hivstatus	Participant HIV status	checkbox, Required<br>2 pcpt_hivstatus___2 HIV Positive
6	send_srv1	Send Survey 1?	checkbox, Required<br>1 send_srv1___1 Yes
7	participants_complete	Section Header: *Form Status*<br>Complete?	dropdown<br>0 Incomplete<br>1 Unverified<br>2 Complete
Instrument: **Information Page** (information_page)			
8	uconn_logo		descriptive

9	info1	Information Sheet for Participation in a Research Study<br><br>UCONN IRB Approved 23-Aug-2019<br><br>Principal Investigator: Seth Kalichman, PhD<br>Title of Study: KISS Surveys<br>Sponsor: National Institute of Mental Health	descriptive
10	info2	You are invited to participate in a research study. This form includes information about the study and contact information if you have any questions.<br><br>The purpose of the KISS Survey is to develop new questionnaires to help us better understand the sexual health experiences of people living in the southeastern USA. You will be asked to complete two electronic surveys on your computer, phone or tablet. You will be asked to complete the surveys on your own, in the privacy of your own space. The surveys contain sensitive and personal questions about your health, personal social experiences, family and social relationships, sex life, use of alcohol and other substances.<br><br>None of this information is stored on your electronic device and your name, or any other identifying information, will not be connected to your surveys. While we hope you will complete every question, but you do have the right to refuse to complete any particular question and you can stop your participation at any time before or during the surveys without any cost or penalty to you.	descriptive
11	info3	Survey 1<br>You will be sent a link for Survey 1 either via a text or email. It will take approximately 30-60 minutes to complete the survey. You will be paid $25 via PayPal or an eGift Card within 24 hours (week days) of completion of the survey. You may select either method of payment.	descriptive
12	info4	Survey 2<br>After you have completed Survey 1 and have been paid, you will receive a second link for Survey 2 via an email or text. It will take approximately 30-60 minutes to complete the survey. You will be paid $35 via PayPal or an eGift within 24 hours (week days) of completion of the Survey 2. You may select either method of payment.	descriptive

13	info5	We ask that you complete each survey as you get them, in a single sitting, meaning that once you start a survey you finish it without leaving it for more than 15 minutes. If the survey is left idle for more than 15 minutes, the link will close and you will need to start from the beginning.<br><br>This study should take less than 3-hours of your time to complete both surveys and all study procedures. Your participation will be anonymous, meaning your name or any other identifying information will not be collected as part or in conjunction with your survey responses.	descriptive
14	info6	You will not be contacted again in the future, unless you expressly wish to know about future surveys and study opportunities. At enrollment, you will be asked to provide your email and phone number in order that you may receive the surveys and eGift cards. The list linking your email and gift card will not be linked to your data. Your email is not connected in any way to your survey responses.<br><br>Some of the questions in the survey are sensitive in nature. As a result, you may become uncomfortable or upset when taking the survey. You can choose to skip any question that you do not want to answer. We ask that you complete the surveys by answering the questions honestly and accurately.<br><br>There is no direct benefit to you from this research. However, the benefits of your participation may impact your community by helping to develop new health and well-being programs for people in your community.	descriptive

652	cov4_pm2<br><br>Show the field ONLY if:<br>[cov3_pm2]=1	How much have heard about CoronaVirus/Covid-19?	radio, Required<br>0 Not much<br>1 Some<br>2 A great deal
653	covknow1_pm2<br><br>Show the field ONLY if:<br>[cov3_pm2]=1	Section Header: *Please answer whether you believe these statements about the new CoronaVirus, also called Covid-19, to be true or false.*<br><br>Antibiotics can cure coronavirus.	radio, Required<br>3 True<br>2 False<br>1 Don't know
654	covknow2_pm2<br><br>Show the field ONLY if:<br>[cov3_pm2]=1	People of all ages can become infected with the coronavirus.	radio, Required<br>3 True<br>2 False<br>1 Don't know
655	covknow3_pm2<br><br>Show the field ONLY if:<br>[cov3_pm2]=1	Coronavirus has been around a long time and only recently came to the USA.	radio, Required<br>3 True<br>2 False<br>1 Don't know
656	covknow4_pm2<br><br>Show the field ONLY if:<br>[cov3_pm2]=1	Eating garlic can lower your chances of getting infected with the coronavirus.	radio, Required<br>3 True<br>2 False<br>1 Don't know
657	covknow5_pm2<br><br>Show the field ONLY if:<br>[cov3_pm2]=1	Coronavirus is no different than a bad case of the flu.	radio, Required<br>3 True<br>2 False<br>1 Don't know
658	covknow6_pm2<br><br>Show the field ONLY if:<br>[cov3_pm2]=1	Most people who are infected with the coronavirus recover from it.	radio, Required<br>3 True<br>2 False<br>1 Don't know
659	covknow7_pm2<br><br>Show the field ONLY if:<br>[cov3_pm2]=1	Antibiotics can be used to prevent infection from the coronavirus.	radio, Required<br>3 True<br>2 False<br>1 Don't know
660	covknow8_pm2<br><br>Show the field ONLY if:<br>[cov3_pm2]=1	The CoronaVirus can be cured with a drug used to treat Malaria.	radio, Required<br>3 True<br>2 False<br>1 Don't know

661	covknow9_pm2<br><br>Show the field ONLY if:<br>[cov3_pm2]=1	The CoronaVirus can be cured with drugs used to treat HIV.	radio, Required<br>3 True<br>2 False<br>1 Don't know
662	cov5_pm2<br><br>Show the field ONLY if:<br>[cov3_pm2]=1	Section Header:<br><br>Do you believe that you have had the CoronaVirus?	radio, Required<br>1 Yes<br>0 No
663	cov6_pm2<br><br>Show the field ONLY if:<br>[cov3_pm2]=1	Have you received a test for CoronaVirus/Covid-19?	radio, Required<br>1 Yes<br>0 No
664	cov6a_pm2<br><br>Show the field ONLY if:<br>[cov3_pm2]=1 and [cov6_pm2]=1	Section Header:<br><br>What was the result of your CoronaVirus/Covid-19 test?	radio, Required<br>1 Positive<br>0 Negative<br>2 Don't know
665	cov_enacted1_pm2<br><br>Show the field ONLY if:<br>[cov6_pm2]=1 and [cov6a_pm2]=1	Section Header:<br><br>In general, how open would you say you are about having tested positive for CoronaVirus?	radio, Required<br>1 Not at all open<br>2 Mostly not open<br>3 Mostly open<br>4 Totally open<br>10 Refuse to answer
666	cov_enacted2_pm2<br><br>Show the field ONLY if:<br>[cov6_pm2]=1 and [cov6a_pm2]=1	How many people did you tell that you tested positive for CoronaVirus?	dropdown, Required<br>0 0<br>1 1<br>2 2<br>3 3<br>4 4<br>5 5<br>6 more than 5
667	cov_enacted3_pm2<br><br>Show the field ONLY if:<br>[cov6_pm2]=1 and [cov6a_pm2]=1	Did you tell people you were sick with something different, so they would not know you had CoronaVirus?	radio, Required<br>1 Yes<br>0 No<br>10 Refuse to answer

668	cov_enacted4_pm2<br><br>Show the field ONLY if: [cov6_pm2]=1 and [cov 6a_pm2]=1	People treated me different when I told them I tested positive for CoronaVirus.	radio, Required<br>5 Strongly Agree<br>4 Somewhat Agree<br>3 Slightly Agree<br>2 Slightly Disagree<br>1 Somewhat Disagree<br>0 Strongly Disagree<br>9 I did not tell anyone I tested positive
669	cov_enacted5_pm2<br><br>Show the field ONLY if: [cov6_pm2]=1 and [cov 6a_pm2]=1	People were supportive when I told them I tested positive for CoronaVirus.	radio, Required<br>5 Strongly Agree<br>4 Somewhat Agree<br>3 Slightly Agree<br>2 Slightly Disagree<br>1 Somewhat Disagree<br>0 Strongly Disagree<br>9 I did not tell anyone I tested positive
670	cov_enacted6_pm2<br><br>Show the field ONLY if: [cov6_pm2]=1 and [cov 6a_pm2]=1	People acted fearful when I told them I tested positive for CoronaVirus.	radio, Required<br>5 Strongly Agree<br>4 Somewhat Agree<br>3 Slightly Agree<br>2 Slightly Disagree<br>1 Somewhat Disagree<br>0 Strongly Disagree<br>9 I did not tell anyone I tested positive
671	time4_pm2	Section Header: *Imagine that you did test positive for CoronaVirus. The next questions ask what you think you would do if you tested positive for CoronaVirus.*<br><br>time	text (datetime_seconds_mdy)<br>Field Annotation: @NOW @HIDDEN-SURVEY
672	cov_antic1_pm2<br><br>Show the field ONLY if: [cov3_pm2]=1 and ([cov 6_pm2]=0 or [cov6_pm 2]=1) and ([cov6a_pm2] =0 or [cov6a_pm2]=2)	How many people do you think you would tell if you tested positive for CoronaVirus?	dropdown, Required<br>0 0<br>1 1<br>2 2<br>3 3<br>4 4<br>5 5<br>6 more than 5

673	cov_antic2_pm2<br><br>Show the field ONLY if: [cov3_pm2]=1 and ([cov6_pm2]=0 or [cov6_pm2]=1) and ([cov6a_pm2]=0 or [cov6a_pm2]=2)	Would you tell people you were sick with something different, so they would not know you had CoronaVirus?	radio, Required<br>1 Yes<br>0 No<br>10 Refuse to answer
674	cov_antic3_pm2<br><br>Show the field ONLY if: [cov3_pm2]=1 and ([cov6_pm2]=0 or [cov6_pm2]=1) and ([cov6a_pm2]=0 or [cov6a_pm2]=2)	People would treat me different if I told them I tested positive for CoronaVirus.	radio, Required<br>5 Strongly Agree<br>4 Somewhat Agree<br>3 Slightly Agree<br>2 Slightly Disagree<br>1 Somewhat Disagree<br>0 Strongly Disagree
675	cov_antic4_pm2<br><br>Show the field ONLY if: [cov3_pm2]=1 and ([cov6_pm2]=0 or [cov6_pm2]=1) and ([cov6a_pm2]=0 or [cov6a_pm2]=2)	People would be supportive if I told them I tested positive for CoronaVirus.	radio, Required<br>5 Strongly Agree<br>4 Somewhat Agree<br>3 Slightly Agree<br>2 Slightly Disagree<br>1 Somewhat Disagree<br>0 Strongly Disagree
676	cov_antic5_pm2<br><br>Show the field ONLY if: [cov3_pm2]=1 and ([cov6_pm2]=0 or [cov6_pm2]=1) and ([cov6a_pm2]=0 or [cov6a_pm2]=2)	People would act fearful if I told them I tested positive for CoronaVirus.	radio, Required<br>5 Strongly Agree<br>4 Somewhat Agree<br>3 Slightly Agree<br>2 Slightly Disagree<br>1 Somewhat Disagree<br>0 Strongly Disagree
677	cov7_pm2<br><br>Show the field ONLY if: [cov3_pm2]=1	Section Header:<br><br>From 0 to 100, how concerned are you about catching the new CoronoaVirus?	slider, Required<br>Slider labels: Not at all concerned, , Extremely Concerned<br>Custom alignment: RH
678	cov8_pm2<br><br>Show the field ONLY if: [cov3_pm2]=1	From 0 to 100, how concerned are you about someone you know catching the new CoronoaVirus?	slider, Required<br>Slider labels: Not at all concerned, , Extremely Concerned<br>Custom alignment: RH
679	cov9_pm2<br><br>Show the field ONLY if: [cov3_pm2]=1	Section Header: *The new CoronaVirus is impacting people in different ways. Have you had any of the following experiences in response to CoronaVirus?*<br><br>Staying indoors and away from public places.	radio, Required<br>2 Yes, a lot<br>1 Yes a little<br>0 No

680	cov10_pm2<br><br>Show the field ONLY if:<br>[cov3_pm2]=1	Canceled plans that involve other people.	radio, Required<br>2 Yes, a lot<br>1 Yes a little<br>0 No
681	cov11_pm2<br><br>Show the field ONLY if:<br>[cov3_pm2]=1	Been unable to get the food you need.	radio, Required<br>2 Yes, a lot<br>1 Yes a little<br>0 No
682	cov12_pm2<br><br>Show the field ONLY if:<br>[cov3_pm2]=1	Been unable to get to a pharmacy because of the new CoronaVirus.	radio, Required<br>2 Yes, a lot<br>1 Yes a little<br>0 No
683	cov13_pm2<br><br>Show the field ONLY if:<br>[cov3_pm2]=1	Been unable to get to medicine you need because of the new virus.	radio, Required<br>2 Yes, a lot<br>1 Yes a little<br>0 No
684	cov14_pm2<br><br>Show the field ONLY if:<br>[cov3_pm2]=1	You cancelled a clinic or doctor.	radio, Required<br>1 Yes<br>0 No
685	cov15_pm2<br><br>Show the field ONLY if:<br>[cov3_pm2]=1	A clinic or doctor closed or cancelled your appointment because of the new CoronaVirus?	radio, Required<br>1 Yes<br>0 No
686	cov16_pm2<br><br>Show the field ONLY if:<br>[cov3_pm2]=1	A service provider of any type closed or cancelled your appointment because of the new CoronaVirus?	radio, Required<br>1 Yes<br>0 No
687	cov17_pm2<br><br>Show the field ONLY if:<br>[cov3_pm2]=1	You asked others to stay away to avoid getting the new Coronavirus.	radio, Required<br>2 Yes, a lot<br>1 Yes a little<br>0 No
688	cov18_pm2<br><br>Show the field ONLY if:<br>[cov3_pm2]=1	You have been asked by others to stay away to protect you from getting the virus.	radio, Required<br>2 Yes, a lot<br>1 Yes a little<br>0 No
689	cov19_pm2<br><br>Show the field ONLY if:<br>[cov3_pm2]=1	Was told not to come to work or school because of the CoronoaVirus.	radio, Required<br>2 Yes, a lot<br>1 Yes a little<br>0 No

690	cov20_pm2<br><br>Show the field ONLY if: [cov3_pm2]=1	Avoided the MARTA/Public Transportation because of the CoronaVirus.	radio, Required<br>2 Yes, a lot<br>1 Yes a little<br>0 No
691	cov21_pm2<br><br>Show the field ONLY if: [cov3_pm2]=1	Section Header: *The Government has taken some actions to prevent the spread of the new CoronaVirus. We are interested in your opinion. There are no right or wrong answers*<br><br>How much do you trust that the Government is doing all it can to prevent the spread of the CoronaVirus?	radio, Required<br>4 Do not Trust at all<br>3 Slightly Trust<br>2 Somewhat Trust<br>1 Trust Completely
692	cov22_pm2<br><br>Show the field ONLY if: [cov3_pm2]=1	How much do you trust information from the CDC about the new CoronaVirus?	radio, Required<br>4 Do not Trust at all<br>3 Slightly Trust<br>2 Somewhat Trust<br>1 Trust Completely
693	cov23_pm2<br><br>Show the field ONLY if: [cov3_pm2]=1	How much do you trust information from the Georgia Department of Public Health about the new CoronaVirus?	radio, Required<br>4 Do not Trust at all<br>3 Slightly Trust<br>2 Somewhat Trust<br>1 Trust Completely
694	cov24_pm2<br><br>Show the field ONLY if: [cov3_pm2]=1	How much do you trust information you are seeing online or in social media about the new CoronaVirus?	radio, Required<br>4 Do not Trust at all<br>3 Slightly Trust<br>2 Somewhat Trust<br>1 Trust Completely
695	time5_pm2	Section Header: *The following ask your opinion regarding the new CoronaVirus. How much do you agree or disagree with each statement? We are interested in your opinion. There are no right or wrong answers.*<br><br>time	text (datetime_seconds_mdy)<br>Field Annotation: @NOW @HIDDEN-SURVEY
696	cov25_pm2<br><br>Show the field ONLY if: [cov3_pm2]=1	It should be a crime for people who know they have the virus but do not take steps to prevent from spreading it.	radio, Required<br>5 Strongly Agree<br>4 Somewhat Agree<br>3 Slightly Agree<br>2 Slightly Disagree<br>1 Somewhat Disagree<br>0 Strongly Disagree<br>10 Refuse to Answer

697	cov26_pm2<br><br>Show the field ONLY if:<br>[cov3_pm2]=1	People who test positive for the new virus should be required to wear identification tags.	radio, Required<br>5 Strongly Agree<br>4 Somewhat Agree<br>3 Slightly Agree<br>2 Slightly Disagree<br>1 Somewhat Disagree<br>0 Strongly Disagree<br>10 Refuse to Answer
698	cov27_pm2<br><br>Show the field ONLY if:<br>[cov3_pm2]=1	I am afraid of the new virus.	radio, Required<br>5 Strongly Agree<br>4 Somewhat Agree<br>3 Slightly Agree<br>2 Slightly Disagree<br>1 Somewhat Disagree<br>0 Strongly Disagree<br>10 Refuse to Answer
699	cov28_pm2<br><br>Show the field ONLY if:<br>[cov3_pm2]=1	People who test positive for CoronaVirus should be quarantined or separated by force from others.	radio, Required<br>5 Strongly Agree<br>4 Somewhat Agree<br>3 Slightly Agree<br>2 Slightly Disagree<br>1 Somewhat Disagree<br>0 Strongly Disagree<br>10 Refuse to Answer
700	cov29_pm2<br><br>Show the field ONLY if:<br>[cov3_pm2]=1	If I tested positive for the CoronaVirus people would treat me differently.	radio, Required<br>5 Strongly Agree<br>4 Somewhat Agree<br>3 Slightly Agree<br>2 Slightly Disagree<br>1 Somewhat Disagree<br>0 Strongly Disagree<br>10 Refuse to Answer

701	cov30_pm2<br><br>Show the field ONLY if:<br>[cov3_pm2]=1	If I tested positive for the CoronaVirus I would not tell anyone.	radio, Required<br>5 Strongly Agree<br>4 Somewhat Agree<br>3 Slightly Agree<br>2 Slightly Disagree<br>1 Somewhat Disagree<br>0 Strongly Disagree<br>10 Refuse to Answer
702	cov31_pm2<br><br>Show the field ONLY if:<br>[cov3_pm2]=1	People who have been to China in the past year should not be allowed into the United States.	radio, Required<br>5 Strongly Agree<br>4 Somewhat Agree<br>3 Slightly Agree<br>2 Slightly Disagree<br>1 Somewhat Disagree<br>0 Strongly Disagree<br>10 Refuse to Answer
703	cov32_pm2<br><br>Show the field ONLY if:<br>[cov3_pm2]=1	I am afraid of people who have this new virus.	radio, Required<br>5 Strongly Agree<br>4 Somewhat Agree<br>3 Slightly Agree<br>2 Slightly Disagree<br>1 Somewhat Disagree<br>0 Strongly Disagree<br>10 Refuse to Answer
704	cov33_pm2<br><br>Show the field ONLY if:<br>[cov3_pm2]=1	Areas in the city that are heavily populated by people from China should be closed off, or 'locked down'.	radio, Required<br>5 Strongly Agree<br>4 Somewhat Agree<br>3 Slightly Agree<br>2 Slightly Disagree<br>1 Somewhat Disagree<br>0 Strongly Disagree<br>10 Refuse to Answer

705	cov34_pm2<br><br>Show the field ONLY if: [cov3_pm2]=1	People who have been to China should be forced to be tested for this new virus.	radio, Required<br>5 Strongly Agree<br>4 Somewhat Agree<br>3 Slightly Agree<br>2 Slightly Disagree<br>1 Somewhat Disagree<br>0 Strongly Disagree<br>10 Refuse to Answer
706	cov35_pm2<br><br>Show the field ONLY if: [cov3_pm2]=1	People from countries with more of the new virus should not be allowed in the US.	radio, Required<br>5 Strongly Agree<br>4 Somewhat Agree<br>3 Slightly Agree<br>2 Slightly Disagree<br>1 Somewhat Disagree<br>0 Strongly Disagree<br>10 Refuse to Answer
707	cov36_pm2<br><br>Show the field ONLY if: [cov3_pm2]=1	People who have been to New York should be forced to be tested for this new virus.	radio, Required<br>5 Strongly Agree<br>4 Somewhat Agree<br>3 Slightly Agree<br>2 Slightly Disagree<br>1 Somewhat Disagree<br>0 Strongly Disagree<br>10 Refuse to Answer
708	cov37_pm2<br><br>Show the field ONLY if: [cov3_pm2]=1	People from states with more of the new virus should not be allowed in the Georgia.	radio, Required<br>5 Strongly Agree<br>4 Somewhat Agree<br>3 Slightly Agree<br>2 Slightly Disagree<br>1 Somewhat Disagree<br>0 Strongly Disagree<br>10 Refuse to Answer
709	time6_pm2	Section Header: *It's common for people to use alcohol and other drugs. Think about yourself when answering the following questions.*<br><br>time	text (datetime_seconds_mdy)<br>Field Annotation: @NOW @HIDDEN-SURVEY

710	audit1_pm2	How often do you have a drink containing alcohol?	radio, Required<br>0 Never<br>1 Monthly or less<br>2 2-4 times a month<br>3 2-3 times a week<br>4 More than 4 times a week
711	audit2_pm2<br>Show the field ONLY if: [audit1_pm2] = '1' or [audit1_pm2] = '2' or [audit1_pm2] = '3' or [audit1_pm2] = '4'	How many drinks containing alcohol do you have on a typical day when you are drinking?	radio, Required<br>0 1 or 2<br>1 3 or 4<br>2 5 or 6<br>3 7 to 9<br>4 10 or more
712	audit3_pm2<br>Show the field ONLY if: [audit1_pm2] = '1' or [audit1_pm2] = '2' or [audit1_pm2] = '3' or [audit1_pm2] = '4'	How often do you have six or more drinks on one occasion?	radio, Required<br>0 Never<br>1 Less than monthly<br>2 Monthly<br>3 Weekly<br>4 Daily or almost daily
713	audit1a_pm2	Did you stop drinking when you tested positive for HIV?	radio, Required<br>1 Yes<br>0 No<br>10 Refuse to answer
714	audit1b_pm2	Did you stop drinking when you started taking HIV medications?	radio, Required<br>1 Yes<br>0 No<br>10 Refuse to answer
715	drugs1_pm2	How often do you use marijuana (pot, grass etc.)?	radio, Required<br>0 Never<br>1 Monthly or less<br>2 2-4 times a month<br>3 2-3 times a week<br>4 More than 4 times a week
